# Secondary Postpartum Hemorrhage Presenting With Bombay Blood Group: A Case Report

**DOI:** 10.7759/cureus.9758

**Published:** 2020-08-15

**Authors:** Likhita Shaik, Abhimanyu Ravalani, Janaki Devara, Sawai Singh Rathore, Romil Singh

**Affiliations:** 1 Internal Medicine, Ashwini Rural Hospital and Research Centre, Solapur, IND; 2 Medical Oncology, Mayo Clinic and Foundation, Rochester, USA; 3 Medicine, Baroda Medical College, Vadodara, IND; 4 Pediatrics, Mayo Clinic, Rochester, USA; 5 Internal Medicine, Dr. Sampurnanand Medical College, Jodhpur, IND; 6 Internal Medicine, Metropolitan Hospital, Jaipur, IND

**Keywords:** bombay blood group, rh-antigen, post-partum, hemorrhage, blood group

## Abstract

Bombay blood group is a rare blood group. Due to its rarity and limitations for transfusions, it is often challenging to manage individuals with Bombay groups in emergencies. Here, we present a case of a 26-year-old woman with the Bombay blood group who had premature rupture of membranes at the 39th week of pregnancy while delivering a male child vaginally. The patient suffered from postpartum hemorrhage due to retention of the placenta and needed an immediate blood transfusion. During the antenatal screening, she was noted to have the O-positive blood group. Cross-matching of her blood was incompatible with O-positive blood and was identified as the Bombay blood group after having tested for anti-H antibodies. The patient underwent transfusion by identifying individuals with the O-positive Bombay blood group. As a result of this, we emphasize the diagnosis and identification of the individuals with the Bombay blood group and make blood available especially in medical emergencies.

## Introduction

Bombay blood group is a rare blood group. Phenotypes of this blood group fail to express H antigen, a precursor of A and B blood group antigens. As these individuals fail to possess antigens on the red blood cell membrane, the serum of these individuals contains anti-H bodies [1,2]. If people with this blood group receive blood transfusions that have H antigens (for example, O blood group), they will be at the risk of acute blood transfusion reaction. These individuals should receive transfusions from the Bombay phenotype or autologous blood [3]. Due to its rarity and limitations, it is very difficult to manage emergencies. Bombay blood group is often misdiagnosed as an O blood group [1]. Postpartum hemorrhage is one of the medical emergencies due to blood loss within 24 hours of delivery (primary postpartum hemorrhage) or from 24 hours of delivery until 12 weeks postdelivery (secondary postpartum hemorrhage). It is very important to maintain hemodynamic stability and need to be managed with blood transfusion [4]. In this case report, we describe a case of Bombay blood group presenting with secondary postpartum hemorrhage. 

## Case presentation

A 26-year-old gravida-three para-two with a history of one miscarriage during the early trimester presented to our hospital at 39 weeks gestation as a case of full-term pregnancy with premature rupture of membranes. She had an uneventful antenatal period, and her most recent antenatal laboratory results at that time showed hemoglobin of 11 mg/dL, blood group status of O, with a positive Rh antigen. Her serum creatinine and blood sugar levels were within normal limits. The patient was negative for the screening of human immunodeficiency virus (HIV), hepatitis B, and syphilis infections. Since immediate delivery was warranted, she was transported to the labor room, and induction of labor was done with intracervical dinoprostone gel. Two hours following induction of labor, she delivered a healthy male child weighing 3.5 kg vaginally, with Apgar scores eight and nine at one and five minutes. 

After the evacuation of the placenta by controlled cord traction, an examination of evacuated placental tissue revealed a missing lobe. This was followed by the development of severe postpartum hemorrhage in the patient, uncontrolled by bimanual massage and oxytocin. Thus, the patient was taken for emergency uterine evacuation of retained placental cotyledon under anesthesia. Laboratory investigations done after the evacuation of the placenta showed a total leucocyte count of 18,000/mm^3 ^(normal range - 4,500 to 11,000/mm^3^), platelet count of 180,000/mm^3 ^(normal range - 150,000 to 400,000/mm^3^), and low hemoglobin of 6.2 mg/dL (normal range - 12.5 to 15.5 mg/dl for women), which indicated a need for packed red cell transfusion.

The patient’s blood was collected and was sent for blood grouping and cross-matching, but was found to be incompatible with multiple units of O-positive blood group. The indirect Coombs test was employed to detect the presence of incomplete antibodies and was found to be positive for the same. Thus, the presence of a rare blood type was suspected, and agglutination with anti-H antisera was performed. Lack of agglutination with anti-H antisera confirmed the diagnosis of the Bombay blood group. 

After this, Bombay blood group O-positive donors were screened rapidly from nearby blood banks, and four units of packed red blood cells and 10 units of fresh frozen plasma were made available. Generally, Bombay blood group units of blood or blood products are sparsely available, but blood banks do maintain the contacts and records of Bombay blood donors with them. The patient was admitted for inpatient monitoring and was transfused with four units of packed red blood cells. In the following week of inpatient care, the patient showed good clinical improvement and was discharged after seven days of admission.

**Figure 1:**
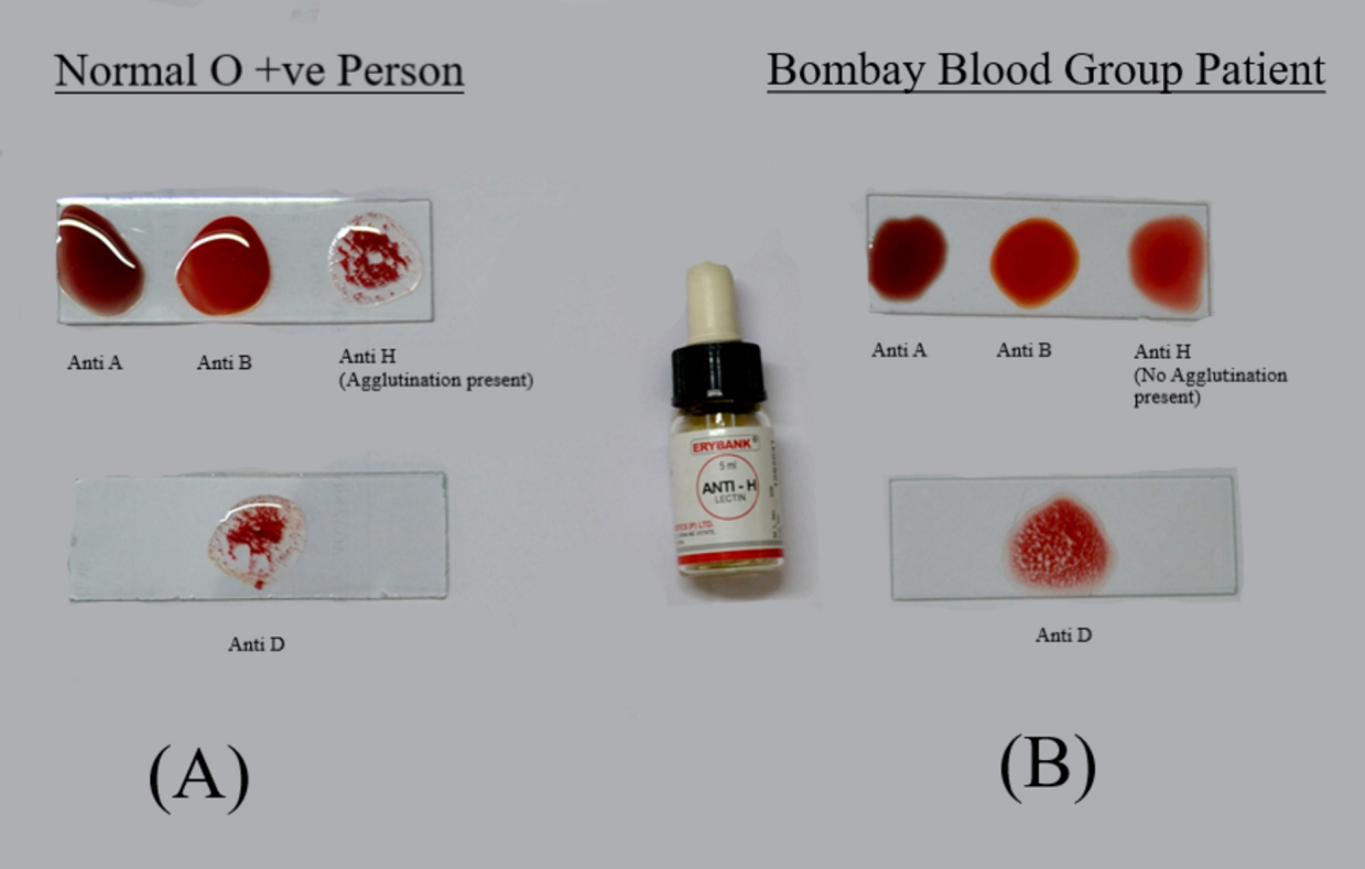
(A) Shows agglutination reaction of the blood sample taken from an individual with an O-positive blood group, showing agglutination with anti-A and anti-B antisera, but no agglutination with anti-D and anti-H antisera. (B) Represents the patient’s blood sample with an O-positive Bombay blood group which shows agglutination with anti-A, anti-B, and anti-H antisera, but no agglutination with anti-D antisera.

The patient again presented to the hospital at 40 days postpartum, with complaints of continuous vaginal bleeding for 15 days with intermittent episodes of excess blood loss, in accordance with the diagnosis of secondary postpartum hemorrhage. The patient was admitted after evidence of retained products of conception in the uterus were detected on ultrasound. The patient’s laboratory investigations showed a serum beta-human chorionic gonadotropin of 2.3 mIU/ml (normal value - less than 5 mIU/ml), hemoglobin of 8.3 mg/dL (normal range - 12.5 to 15.5 mg/dl for women), total leucocyte count of 6,300/mm^3 ^(normal range - 4,500 to 11,000/mm^3^​​​​), and a platelet count of 360,000/mmm^3 ^(normal range - 150,000 to 400,000/mm^3^). The difference in agglutination reaction between normal O-positive blood group patient and Bombay blood group patient is shown in Figure 1. 

**Figure 2:**
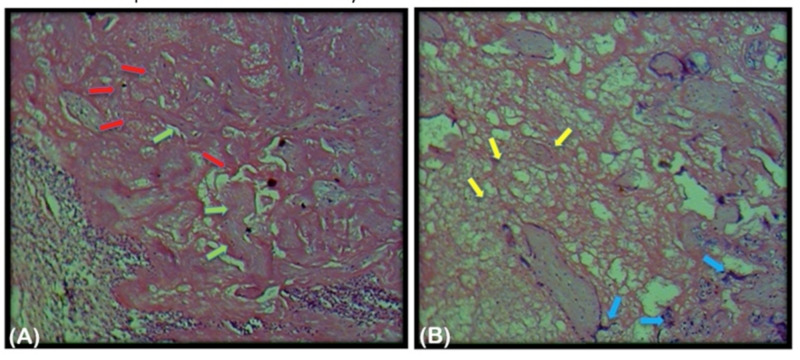
Slides (A) and (B) are the histopathology slides of the placental tissue evacuated from the patient’s uterus. The red and green arrows in slide (A) show degenerated villi with hyalinization and villous stromal fibrosis, respectively. The blue and yellow arrows in slide (B) show the presence of syncytial knots and evidence of fibrinoid necrosis, respectively.

The patient was scheduled for evacuation and curettage, and a rapid search for the Bombay blood group was done. Preoperatively, two units of packed red blood cells were made available on an urgent basis from various Bombay blood group donors as per indication of transfusion. The patient then underwent repeat uterine evacuation and curettage under ultrasonography guidance uneventfully, followed by inpatient admission for monitoring. The evacuated placental tissue was sent for histopathology examination, which revealed degenerated chorionic villi, trophoblastic cells, and fibrinoid material. The patient was discharged on the fifth day postoperatively after a repeat ultrasound was done to confirm the complete evacuation of conception products inside the uterine cavity. The histopathology of the placental tissue from the patient's uterus is shown in Figure 2. 

## Discussion

The Bombay blood group phenotype was first identified by Bhende in Bombay, India, in 1952 [5]. With a prevalence rate of 1:10,000 in India and 1:1,000,000 in Europe, the Bombay blood group phenotype is very rare [2]. It is also rare in prevalence, with a frequency of 1:250,000 in Caucasians [1].

The Bombay blood group exists due to point H gene mutation with a mutant variety being the H gene, which does not encode any protein. Therefore, the protein called fucosyltransferase, which is coded by the H gene, is deficient. This protein mediates the incorporation of L-fucose into the H-antigen parent chain. Thus, due to the absence of this protein, the H antigen cannot be made. Since the H antigen on red blood cells is the precursor of A and B antigens, these lead to the cumulative absence of A and B antigens. All these deficiencies lead to the production of anti-A, anti-B, and anti-H antibodies. Lack of antigens A and B simulates the blood group O. Furthermore, anti-H antibodies induce cross-reactions with all forms of blood groups, including the O blood group bearing the H antigen. Therefore, to prevent mismatched blood transfusion, these patients can only obtain blood from a person carrying a Bombay blood group type [6].

People with the Bombay blood group phenotype are often misdiagnosed as the O blood group in cell typing. These individuals produce an acute hemolytic transfusion reaction if they receive blood group O or any other blood group besides the Bombay blood group. It is due to the carriage of strong anti-H in their plasma. Acute hemolysis may result in disseminated intravascular coagulation or acute renal failure, resulting in significant morbidity and mortality, notably in unconscious patients who might have received large portions of mismatched blood before manifestations of these hemolytic reactions emerge [7]. 

In patients with the Bombay blood group, the key therapeutic obstacle is to arrange cross-matched blood due to its very low occurrence in the population. Our patient was managed with the required transfusion by identifying individuals with the O-positive Bombay blood group.

Ultimately, it can be asserted that just blood grouping is not enough before blood transfusion. Cross-matching is crucial to acknowledge this rare blood group. To validate the diagnosis, reverse grouping or plasma grouping should be done. 

## Conclusions

When undetected, the Bombay blood group can cause fatal hemolytic transfusion reactions or severe complications from hemorrhage. Individuals with the Bombay blood group in emergencies need to be approached meticulously. For this reason, it is very important to identify this blood group by routine serum typing to identify anti-H antibodies in O-positive maternal blood. We recommend implementing this practice during routine antenatal screening to prevent complications during pregnancy or delivery. 
